# Cigarette, waterpipe and e-cigarette use among an international sample of medical students. Cross-sectional multicenter study in Germany and Hungary

**DOI:** 10.1186/s12889-018-5494-6

**Published:** 2018-05-03

**Authors:** Erika Balogh, Nóra Faubl, Henna Riemenschneider, Péter Balázs, Antje Bergmann, Károly Cseh, Ferenc Horváth, Jörg Schelling, András Terebessy, Zoltán Wagner, Karen Voigt, Zsuzsanna Füzesi, István Kiss

**Affiliations:** 1 Department of Public Health Medicine, University of Pécs Medical School, Szigeti str 12, Pécs, 7624 Hungary; 2 Department of Behavioral Sciences, University of Pécs Medical School, Szigeti str 12, Pécs, 7624 Hungary; 3 Department of General Practice, Medical Clinic 3, University Hospital Carl Gustav Carus, Technische Universität Dresden, Fetscherstr. 74, 01307 Dresden, Germany; 4 Department of Public Health, Faculty of Medicine, Semmelweis University Budapest, Nagyvárad tér 4, Budapest, 1089 Hungary; 5 Department of General and Family Medicine, Medical Faculty, Ludwig-Maximilians-Universität München, Pettenkoferstr. 8a, 80336 Munich, Germany

**Keywords:** Cigarette smoking, Waterpipe/shisha/hookah, E-cigarette, Socio-cultural factors, Medical students, Multicenter study

## Abstract

**Background:**

Tobacco use is the leading preventable cause of death worldwide. Besides cigarette smoking, waterpipe and e-cigarettes are gaining popularity among young adults. Medical students’ smoking behavior is of particular interest because of their impending role in health promotion as future physicians. Aim of our study is to examine the prevalence and predictors of cigarette, waterpipe and e-cigarette use and the association of tobacco use with self-reported health status in an international sample of medical students.

**Methods:**

In a multicenter cross-sectional study data on different aspects of health behavior were collected from medical students of 65 nationalities using a self-administered questionnaire in Germany (Dresden, Munich) and Hungary (Budapest, Pécs). The survey was conducted among 1st, 3rd and 5th year students. To explore associations between smoking behavior and socio-cultural factors Pearson’s chi^2^-tests and multivariate binary logistic regression analyses were performed.

**Results:**

The largest subpopulations were formed by German (*n* = 1289), Hungarian (*n* = 1055) and Norwegian (*n* = 147) students. Mean age was 22.5 ± 3.3 years. Females represented 61.6% of the sample. In the whole sample prevalence of cigarette smoking was 18.0% (95% CI 16.6–19.4%), prevalence of waterpipe use was 4.8% (95% CI 4.0–5.7%), that of e-cigarette 0.9% (95% CI 0.5–1.2%). More males (22.0%) than females (15.5%) reported cigarette smoking. The lowest prevalence of cigarette smoking was found among Norwegian students (6.2%). Cigarette smokers were older, waterpipe users were younger than non-users. E-cigarette use was not associated with age of the students. Religious involvement was protective only against cigarette smoking. Financial situation showed no association with any kind of tobacco consumption. Cigarette smokers and e-cigarette users were less likely to report very good or excellent health status.

**Conclusions:**

Cigarette smoking is still the most popular way of consuming tobacco, although alternative tobacco use is also prevalent among medical students. To further health consciousness, medical schools should pay more attention to students’ health behavior, especially their smoking habits. Tobacco prevention and cessation programs for medical students should consider not only the health risks of cigarette smoking but the need to discourage other forms of tobacco use, such as waterpipe.

## Background

Despite numerous efforts to stop the tobacco epidemic, tobacco use is still the single most preventable cause of morbidity and mortality worldwide [1]. This risk factor is responsible alone for the death of more than seven million people across the world each year [2]. Among regions of the World Health Organization, Europe has the highest prevalence of tobacco smoking in the adult population and some of the highest prevalence of tobacco use by adolescents [3]. Health professionals, especially physicians, have a unique role while fighting tobacco smoking. Besides providing smoking cessation plans to their patients, they are role models for society at large. Since physicians who smoke are less likely to advise their patients to quit [4, 5], the smoking behavior of future physicians (i.e. medical students) does influence not only their personal health but also the health of their future patients.

Previous studies have detected differences in smoking prevalence among medical students with regard to gender and country of origin [6–8], however they did not consider socio-economic status and religious involvement which are known to influence smoking behavior in young adults [9–11].

New and emerging tobacco products, waterpipe and e-cigarette use are gaining popularity among the youth and young adults worldwide including dual waterpipe and cigarette use [12, 13]. Waterpipe smoking is believed by many users to be less detrimental to health than traditional cigarette consumption [14]. On the contrary, accumulating evidence shows that waterpipe smoking is harmful and addictive [15]. There is not yet enough evidence on the safety of e-cigarettes. Although electronic nicotine delivery systems release generally fewer toxic chemicals than traditional cigarettes, and are less harmful than cigarettes, their use among youth and young adults is far from being safe. E-cigarettes may trigger nicotine dependence [16]. Though cigarette smoking has been studied repeatedly among medical students in Europe [6–8, 17–20], data is limited on waterpipe and e-cigarette use in this specific population [21, 22].

### Aim of the study

The aim of this study was to assess and compare the prevalence of cigarette, waterpipe and e-cigarette use in an international sample of medical students at German and Hungarian universities and to investigate associations between tobacco use and a series of socio-demographic characteristics (gender, age, academic year, nationality, religiosity and financial situation). In order to examine whether smoking has an effect on the current health status of future physicians (i.e. medical students), the relationship between self-reported health status and tobacco use was also analyzed. Monitoring the prevalence of traditional and emerging forms of tobacco use among medical students is essential to detect any changes in tobacco consumption patterns, which can better help tailor smoking cessation policies to this subpopulation.

## Methods/design

### Study design and survey instrument

Our cross-sectional multicenter study used a self-administered questionnaire and was carried out at four Medical Faculties in Germany and Hungary (Technische Universität Dresden, Ludwig-Maximilians-Universität Munich, Semmelweis University Budapest and University of Pécs). The questionnaire development process and piloting of the study among medical students from different nationalities has been reported elsewhere [23].

The questionnaire was based largely on selected items of validated instruments (e.g. 36-Item Short Form Survey Instrument [24]) and previous studies from Technische Universität Dresden and Semmelweis University [6, 25]. The questionnaire was first developed in English, and then translated into German and Hungarian. Beyond socio-demographic characteristics and self-reported health status, data on several risk behaviors, including tobacco consumption, were recorded. This paper focuses on tobacco use and its socio-demographic correlates.

Participants smoking cigarettes at least once a month were classified as current cigarette smokers, those who smoked cigarettes every day as daily smokers, current cigarette smokers who did not smoke every day as occasional smokers. Students using waterpipe or e-cigarette at least once a month were considered waterpipe users or e-cigarette users, respectively. Religious involvement was assessed with the question: “Do you consider yourself as religious?”. Answer choices included “not at all”, “not very”, “moderate”, “very” and “no opinion”. Self-reported health status was categorized as “excellent”, “very good”, “good”, “fair” or “poor”. Participants were also asked to rate their financial situation on a scale from 1 (no financial problems) to 5 (everyday financial problems).

### Study participants and setting

In 2014, the total population of registered 1st, 3rd and 5th year medical students (*n* = 5223) at the four study sites were invited to participate in our survey. Participation was anonymous and voluntary. Consent to participate was given by responding to the questionnaire as stated on its cover letter. The study was carried out during mandatory seminars/tutorials and lectures using the same non-random sampling method for all three year groups as follows: questionnaires were distributed to those attending seminars or lectures (thus students who were absent from these sessions did not have the opportunity to participate in the survey). All questionnaires, even blank ones, were collected in boxes at the doors to allow non-respondents to remain anonymous. The response rate was calculated by dividing the number of completed or partially completed questionnaires by the number of all registered 1st (*n* = 2545), 3rd (*n* = 1345) and 5th year (*n* = 1333) medical students. The study protocol was reviewed and approved by appropriate institutional review boards.

As reported in a previous publication on German medical students’ smoking behavior, prevalence of cigarette smoking was the same in Germany and abroad (Pécs and Budapest, Hungary) [26], thus the German students’ data were merged in the current analysis. Medical faculties in Budapest and Pécs offer medical degree programs in Hungarian, German and English language. As a result, approximately 40% of students are from abroad and a large group of international students were therefore included in the survey. German, Hungarian and Norwegian students were the largest subpopulations. Data of all other students were collapsed and referred to as multinational group.

### Statistical analysis

Analysis was performed using IBM-SPSS v.20. Prior to statistical analysis, religious involvement was transformed into a dichotomized variable using the same categories as those used by Yong et al. [27] into a group with high religiosity (“very religious”) and a group with all other students. To be used in binary logistic regression analyses, health status was converted into a binary variable: students with excellent or very good health status versus all other students. Financial situation was used in four different dichotomized forms (1 versus 2–5; 1–2 versus 3–5; 1–3 versus 4–5; 1–4 versus 5) to identify relevant associations between tobacco use and economic status. An independent samples *t*-test was used for comparing means of metric data. Pearson’s chi^2^-tests were performed to compare nominal variables and to examine univariate associations between socio-cultural factors and smoking behavior. *Z*-tests were carried out with Bonferroni correction to assess differences in the prevalence of cigarette, waterpipe smoking, and e-cigarette use among subgroups of different nations. Multivariate binary logistic regression was conducted to investigate the effect of multiple factors on cigarette, e-cigarette and waterpipe smoking, and on self-reported health status. Students who could not be classified as in the 1st, 3rd or 5th academic years were not included in the analyses examining the association between smoking status and study year.

## Results

A total of 2935 medical students from 65 different nations (response rate: 56.2%) at four universities participated in this multicenter study. Gender was reported in 2925 cases. Socio-demographic characteristics of the participants are shown in Table 1. German, Hungarian and Norwegian students were the most represented nationalities in the sample; the remaining students formed the multinational group. Among 324 German and 147 Norwegian students studying in Hungary only two (0.4%) specified Hungary as country of origin. Most students in the multinational group (89.6%) reported that the country in which they had spent the most of their life was one other than the country of their current medical school. These findings show that most of the students of other nationalities were temporary residents in Germany and Hungary. The composition of the multinational group was heterogeneous including students from European (54.6%), Asian (32.2%), American (7.1%) and African (6.1%) countries. Data on cigarette smoking was available in 2883 cases, waterpipe use in 2725, and e-cigarette use in 2688 cases. Proportion of cases with missing data on cigarette, waterpipe and e-cigarette use was less than 10%.

**Table 1:** Sample characteristics (*n* = 2925)

Characteristic	Statistic
Age, mean ± SD	22.5 ± 3.3
Gender: female, n (%)	1803 (61.6)
Nationality	
German, n (%)	1289 (44.1)
Hungarian, n (%)	1055 (36.1)
Norwegian, n (%)	147 (5.0)
Multinational group, n (%)	434 (14.8)
Academic year	
First, n (%)	1252 (42.8)
Third, n (%)	889 (30.4)
Fifth, n (%)	666 (22.8)
Health status: very good or excellent, n (%)	1935 (66.4)
Financial situation on a scale of five	
1 (no financial problems), n (%)	1227 (42.5)
2, n (%)	835 (28.9)
3, n (%)	579 (20.0)
4, n (%)	174 (6.0)
5 (everyday financial problems), n (%)	44 (1.5)
Religion	
Roman catholic, n (%)	1002 (34.3)
Evangelical/Lutheran, n (%)	398 (13.6)
Religiosity: very religious, n (%)	328 (11.2)

*SD* Standard deviation

### Cigarette smoking

Overall the prevalence of cigarette smoking was 18.0% (95% confidence interval [CI] 16.6–19.4%). Significantly more males smoked cigarettes than females. Gender differences of smoking rates were statistically significant in the subgroups of German and Hungarian students. Prevalence of cigarette smoking was the highest in the multinational group and it was the lowest among Norwegian students (Table 2). None of the Norwegian students were daily smokers (Table 3).

**Table 2:** Prevalence of cigarette smoking by gender and nationality

	Cigarette smokers			
	Total	Males	Females	Difference between genders^a^
Total sample n = 2883	518 (18.0)	242 (22.0)	276 (15.5)	*p* < 0.001
German students n = 1269	211 (16.6)	100 (20.2)	111 (14.3)	*p* = 0.006
Hungarian students n = 1042	198 (19.0)	88 (23.7)	110 (16.4)	*p* = 0.004
Norwegian students *n* = 145	9 (6.2)^b^	3 (5.8)^c^	6 (6.5)^d^	*p* = 0.870
Multinational group *n* = 427	100 (23.4)^e^	51 (27.9)	49 (20.1)	*p* = 0.060
Difference among nationalities^a^	*p* < 0.001	p = 0.004	*p* = 0.012	

Data expressed as n (%), ^a^Pearsons chi^2^-test, ^b,c,d,e^*Z*-test with Bonferroni correction: ^b^*p* < 0.05 versus German, Hungarian and multinational groups, ^c^*p* < 0.05 versus Hungarian and multinational groups, ^d^*p* < 0.05 versus multinational group, ^e^*p* < 0.05 versus German and Norwegian groups

**Table 3:** Prevalence of daily cigarette smoking by gender and nationality

	Daily cigarette smokers			
	Total	Males	Females	Difference between genders^a^
Total sample *n* = 2883	220 (7.6)	115 (10.4)	105 (5.9)	*p* < 0.001
German students *n* = 1269	91 (7.2)	47 (9.5)	44 (5.7)	*p* = 0.010
Hungarian students *n* = 1042	78 (7.5)	38 (10.2)	40 (6.0)	*p* = 0.013
Norwegian students *n* = 145	0 (0.0)^b^	0 (0.0)^c^	0 (0.0)^c^	n.a.
Multinational group *n* = 427	51 (11.9)	30 (16.4)	21 (8.6)	*p* = 0.014
Difference among nationalities^a^	*p* < 0.001	*p* = 0.004	*p* = 0.028	

Data expressed as n (%), ^a^Pearsons chi^2^-test, ^b,c^*Z*-test with Bonferroni correction: ^b^p < 0.05 versus German, Hungarian and multinational groups, ^c^*p* < 0.05 versus multinational group, n.a. not analyzed

About half of the daily smokers consumed 10 or more cigarettes a day (interquartile range [IR]: 5–12 cigarettes/day). Occasional smokers consumed an average of 4.7 cigarettes on one occasion (IR: 2–5 cigarettes/occasion). The majority (70.0%) of daily smokers and almost the half (45.0%) of occasional smokers had already tried to quit smoking.

Cigarette smokers had higher mean age than non-smokers (23.8 ± 3.4 years versus 22.4 ± 3.2 years, *t*-test, *p* < 0.001). Age difference between smokers and non-smokers was only significant in the 1st academic year. There was no age difference between smokers and non-smokers in the 3rd an 5th study years.

There were fewer cigarette smokers among students who reported being “very religious” compared to those not being very religious (13.0% versus 18.6%, chi^2^-test, *p* = 0.013). According to the chi^2^-tests, prevalence of current cigarette smoking was not related to the students’ financial situation regardless how it was dichotomized prior to analysis (regardless of the cut-off chosen for dichotomization). Only 7.6% reported poor financial circumstances (4–5 on a scale of five) and 92.4% saw their financial status as very good, good or average (1–3 on a scale of five).

According to a multivariate binary logistic regression analysis, cigarette smoking was significantly related to age, gender, nationality, study year and religiosity (Nagelkerke R^2^ = 0.044). Male gender, older age, nationality “other than Norwegian” were associated with increased odds for cigarette smoking, whereas high religiosity and studying in the 5th academic year were protective (Table 4).

**Table 4:** Predictors of cigarette smoking status based on multivariate binary logistic regression

	*P*-value	Exp(B)	95% confidence interval Exp(B)	
Cigarette smoking status: current smoker			Lower	Upper
Constant	< 0.001			
Age (per year increase)	< 0.001	1.085	1.050	1.122
Male gender (ref. female)	0.001	1.420	1.163	1.734
Nationality (ref. Norwegian)				
German	0.004	2.800	1.394	5.623
Hungarian	< 0.001	4.097	2.031	8.265
Multinational group	< 0.001	4.542	2.215	9.316
Academic year (ref. first)				
Third	0.119	0.825	0.647	1.051
Fifth	0.010	0.683	0.512	0.911
Very religious (ref. all other)	0.007	0.614	0.431	0.876

### Waterpipe smoking

Prevalence of waterpipe smoking was 4.8% (95% CI 4.0–5.7%). Most of the waterpipe smokers (77%) used waterpipe 1–3 times a month, 11% once a week, 9% several times a week, and only 3% daily. Mean age of waterpipe users was lower than that of non-users (21.5 ± 2.9 years versus 22.6 ± 3.2 years, *t*-test, *p* < 0.001). Waterpipe use was more common among male students than among females. Gender difference was statistically significant among German students and in the multinational group (Table 5). None of the female students were daily users, whereas 0.4% of males used waterpipe every day. No statistically significant differences were found in the prevalence of waterpipe smoking as related to nationality, financial situation or religious involvement. Association of age and gender with waterpipe tobacco use remained significant after adjusting for each other, nationality, study year and religiosity (Table 6, Nagelkerke R^2^ = 0.053).

**Table 5:** Prevalence of waterpipe smoking by gender and nationality

	Waterpipe smokers			
	Total	Males	Females	Difference between genders^a^
Total sample *n* = 2725	132 (4.8)	73 (7.0)	59 (3.5)	*p* < 0.001
German students *n* = 1207	51 (4.2)	30 (6.4)	21 (2.9)	*p* = 0.003
Hungarian students *n* = 996	57 (5.7)	27 (7.5)	30 (4.7)	*p* = 0.067
Norwegian students *n* = 138	4 (2.9)	1 (2.0)	3 (3.4)	*p* = 0.656
Multinational group *n* = 384	20 (5.2)	15 (8.9)	5 (2.3)	*p* = 0.004
Difference among nationalities^a^	*p* = 0.271	*p* = 0.353	*p* = 0.208	

Data expressed as n (%). ^a^Pearsons chi^2^-test

**Table 6:** Predictors of waterpipe smoking status based on multivariate binary logistic regression

	*P*-value	Exp(B)	95% confidence interval Exp(B)	
Waterpipe smoking status: smoker			Lower	Upper
Constant	0.687			
Age (per year increase)	0.008	0.877	0.796	0.966
Male gender (ref. female)	< 0.001	2.097	1.451	3.031
Nationality (ref. Norwegian)				
German	0.938	0.959	0.334	2.753
Hungarian	0.501	1.437	0.499	4.135
Multinational group	0.574	1.377	0.452	4.194
Academic year (ref. first)				
Third	0.439	0.830	0.517	1.331
Fifth	0.435	0.763	0.387	1.505
Very religious (ref. all other)	0.076	0.515	0.247	1.071

### E-cigarette use

Prevalence of e-cigarette use was 0.9% (95% CI 0.5–1.2%) with only 12 daily users (0.4%). More students in the multinational group used e-cigarettes as compared to the German and Hungarian groups. E-cigarette use was not related to age, gender, study year, religiosity or financial situation. Multivariate binary logistic regression confirmed the association of e-cigarette use and nationality (Table 7, Nagelkerke R^2^ = 0.11).

**Table 7:** Predictors of e-cigarette use based on multivariate binary logistic regression

	*P*-value	Exp(B)	95% confidence interval Exp(B)	
E-cigarette smoker status: smoker			Lower	Upper
Constant	0.045			
Age (per year increase)	0.773	0.978	0.842	1.137
Male gender (ref. female)	0.139	1.865	0.817	4.257
Nationality (ref. Multinational group)				
Hungarian	< 0.001	0.080	0.022	0.292
German	< 0.001	0.173	0.068	0.441
Norwegian	0.162	0.231	0.030	1.802
Academic year (ref. first)				
Third	0.919	0.947	0.328	2.730
Fifth	0.334	1.781	0.552	5.746
Very religious (ref. all other)	0.095	2.381	0.859	6.601

### Association of cigarette smoking with waterpipe and e-cigarette use

Waterpipe and e-cigarette use were more common among cigarette smokers than non-smokers (Table 8). Based on binary logistic regression models cigarette smoking was associated with a 2.8-fold odds for waterpipe use (exponentiation of the B coefficient, [Exp(B)] = 2.794, 95% CI 1.879–4.155, *p* < 0.001, Nagelkerke R^2^ = 0.081), and with an almost 9-fold odds for e-cigarette use (Exp(B) = 8.739, 95% CI 3.558–21.465, *p* < 0.001, Nagelkerke R^2^ = 0.201) when compared to non-smoking, even after adjusting for age, gender, nationality, study year and religiosity.

**Table 8:** Association of cigarette smoking with waterpipe and e-cigarette use

	No waterpipe	Waterpipe	Chi^2^-test	No e-cigarette	E-cigarette	Chi^2^-test
*Cigarette non-smoker*			*p* < 0.001			*p* < 0.001
N	2108	77		2146	8	
% of total sample %	78.2	2.9		80.6	0.3	
of non-smoker	96.5	3.5		99.6	0.4	
*Cigarette smoker*						
N	460	52		491	16	
% of total sample %	17.1	1.9		18.5	0.6	
of smoker	89.8	10.2		96.8	3.2	

### Association of cigarette and e-cigarette smoking with health status

The proportion of medical students reporting very good or excellent health status was lower among cigarette smokers compared to non-smokers (58.9% versus 68.1%, chi^2^-test, *p* < 0.001), and also among e-cigarette users compared to non-users (37.5% versus 66.8%, chi^2^-test, *p* = 0.002).

This negative impact of cigarette smoking and e-cigarette smoking on self-reported health status remained significant even after adjusting for each other, age, gender, nationality, study year and religiousness according to a logistic regression model (Nagelkerke R^2^ = 0.067): the probability of reporting very good or excellent health status was almost 30% lower among cigarette smokers compared to non-smokers (Exp(B) = 0.717, 95% CI 0.579–0.888, *p* = 0.002), and 70% lower among e-cigarette users compared to non-users (Exp(B) = 0.300, 95% CI 0.126–0.715, *p* = 0.007). No such relationship between self-reported health status and waterpipe smoking was detected in our sample.

## Discussion

Marked variation in smoking prevalence was observed among medical students by gender and nationality. Our findings of male predominance in smoking rates corresponds with previous studies in general populations [28, 29], as well as with specific studies of medical students [6, 8, 17].

Overall, the prevalence of smoking among medical students in our cross-sectional study was lower than that of the comparable young adult populations in Germany, Hungary and Norway. This is a favorable trend as compared to a previous European survey that found the smoking prevalence to be higher among medical students than the general population [8]. In our study, prevalence of cigarette smoking was 20% among German male and 14% among German female students, whereas smoking prevalence was 31% among German males and 21% among German females aged 18–24 years as reported by the 2015 Epidemiological Survey of Substance Abuse [30]. According to the European Health Interview Survey 2014, smoking rates in Hungary were 42% in males and 29% in females in the 18–34 age group [29], which were higher than prevalence of smoking among Hungarian medical students in our study (24% in males, 16% in females). Norway had relatively low smoking rates in 2014 in the young adult population (20% in males and 14% in females aged 16–24 years [31]). Accordingly, we found very low smoking rates among Norwegian medical students (6.5% in females and 5.8% in males). Moreover, none of the Norwegian students were daily smokers, while about 7% of German and Hungarian students smoked cigarettes every day.

Although it cannot be answered directly from our results, knowledge about health risks of smoking as well as personal beliefs about roles as future physicians may have contributed to the lower smoking prevalence among medical students as compared to the general population. Based on European studies, the majority of medical students believed that health professionals are role models for patients [8], and almost 70% believed to know the health risks of smoking [19]. It is also possible that other factors such as parental smoking and peer influences may explain the difference in smoking rates between medical students and the general population.

Prevalence of cigarette smoking among medical students reported by former studies at German universities (males 22–42%, females 13–22% [6, 8, 17]) was similar to or higher than that of German medical students in our study (males 20%, females 14%). Previous surveys among Hungarian medical students showed wide variation in the prevalence of smoking (from 18 to 40% [18, 20]); our study mirrors those with the lower smoking rate. Surveys of Norwegian medical students found that smoking prevalence was 20–29% among males and 8–19% among females [7, 32], whereas in our study only 5.8% of Norwegian male and 6.5% of Norwegian female students were current smokers. Summarizing the existing data on smoking rates among medical students, the most obvious difference between previously published results and our findings was observed in Norwegian students which showed a remarkable decrease in the prevalence of cigarette smoking.

Taken together, similar differences in smoking rates could be found between nationalities both in the general population and among medical students. Norway had the lowest smoking rates in both settings and an ongoing decrease in the prevalence of cigarette smoking among Norwegian young people has been reported recently [33]. Lower prevalence of cigarette smoking in Norway may be explained by Norway’s long standing ban on all tobacco advertising, other preventive and control measures against tobacco use, and partly by a recent shift to snuff use [34].

In the last decades several tobacco control measures were introduced in Germany and Hungary as well. In Germany, numerous measures (increases in cigarette taxes, raising the minimum age for purchasing tobacco products, ban on advertising, laws on the protection of non-smokers and warnings on cigarette packages) have been implemented to reduce tobacco consumption. Nevertheless, there is still room for improvement in many areas of tobacco control policy [35]. According to the results of the 2013 Tobacco Control Scale, in which 34 European countries were compared with regard to their efforts to achieve effective tobacco prevention and control, Germany took the 33th place [36]. In recent years Hungary has also adopted or strengthened a series of tobacco control measures. The most important of these are the indoor smoking ban in public places and some outdoor prohibitions, the significant tax increase on cigarettes, the combined warnings (text and pictures) on cigarette packages, and the drastic reduction in the number of stores selling tobacco products [37]. Thanks to these efforts, Hungary has been ranked above average (11th place) on the European tobacco control scale in 2013 [36]. In that year, Norway ranked fourth on the same scale [36]. Although a causal relationship between a country’s tobacco strategy and smoking rates among its medical students cannot be established, the promotion of a comprehensive smoke-free legislation with a consequent social rejection of smoking may contribute to a decrease in smoking rates among medical students.

In our sample about 5% of the respondents were current waterpipe smokers. An even higher prevalence of current waterpipe smoking (11%) was reported in a previous study involving medical students in London [21]. The lack of awareness about the harmful health effects of waterpipe smoking, the social nature of its use and the presence of waterpipe cafés near educational establishments may contribute to the growing use of waterpipe among medical students [12, 21]. In our study, the majority of waterpipe smokers used tobacco in this way only 1–3 times a month, which is lower than the frequency shown to induce nicotine dependence. Median waterpipe smoking frequency was 6 days per month when the first symptoms of nicotine dependence emerged [38]. Nevertheless, concurrent use of waterpipe and cigarettes, which was reported by about 50 students (1.9%) in our study, increases the risk for nicotine exposure and other smoking related health effects.

Prevalence of e-cigarette smoking was relatively low (0.9%) in our sample, even lower than that (3.5%) found in a recent study at the Medical University of Silesia, Poland [22]. However, data concerning prevalence of e-cigarette use should be interpreted with caution because of the low number of e-cigarette users. Further studies are required to monitor e-cigarette use among medical students.

In the entire sample, prevalence of cigarette smoking was lower in students who reported high religiosity. Religious involvement was preventive in other populations as well [10, 11]. There is evidence that religiosity is associated with a lower prevalence of smoking initiation [11]. Furthermore, it has been demonstrated that very religious smokers were more likely to be interested in quitting within the next 6 months than their less religious counterparts [27]. However, Bailey et al. found that religiosity was not associated with successful smoking cessation in middle-aged adults [11].

Tobacco use was not related to students’ financial status, perhaps because most respondents reported a stable financial situation. Of course, our results may not be generalizable to other populations with a wider range of socio-economic backgrounds.

Although the whole sample had a narrow age range, a statistically significant difference in age between smokers and non-smokers could be detected. Cigarette smokers had an older mean age than non-smokers while prevalence of smoking was not higher in students further along in their studies. This apparent contradiction may be explained by the finding that the age difference between smokers and non-smokers was only significant in the 1st academic year, which had the largest sample size among study years, leading to a significant difference in mean age between smokers and non-smokers of the whole sample. Thus, it seems that being older than peers in the same class cohort, rather than age, was associated with cigarette smoking. Presumably, students who were older than their peers may have started university later or repeated a grade. However, the exact reason for the age difference was not investigated in the present study.

Cigarette smokers were older than non-smokers, whereas waterpipe users were younger than non-users. Similar findings concerning waterpipe smoking and age have been shown previously in the general European population as well: the highest prevalence of waterpipe tobacco use was detected among the youngest respondents [39]. Further studies are needed to determine whether this trend will herald an increase in waterpipe use and a decrease in cigarette smoking among next generations of medical students.

Cigarette smoking was associated with worse self-reported health status in our study. Cigarette smokers were less likely to rate their health status as very good or excellent, which is in line with previous observation of lower health-related quality of life scores in smoking university students compared to non-smokers [40]. These findings are alarming given the young age of the respondents. An even stronger negative association between self-reported health status and e-cigarette use was found. Slightly more than one-third of the e-cigarette users considered their health status to be very good or excellent. Further research is needed to confirm and clarify this finding. On the other hand, waterpipe use did not affect self-reported health status, probably because the majority of waterpipe smokers used this form of tobacco only 1–3 times a month.

Young smokers may recognize their addiction to tobacco when they experience withdrawal symptoms during their first attempt to quit [41]. In our study 288 young cigarette smokers (56% of current smokers) have already tried to quit smoking, but failed. Smokers who had one or more recent failed cessation attempts are less likely to quit successfully [42]. Furthermore, young adults tend to underutilize evidence-based cessation treatments, although all interventions effective for the general adult population are also effective for young adults [43].

University years provide a unique opportunity for interventions to change harmful health behaviors of future physicians, which is of particular interest not only for improving their personal health, but also because of their impending role in health promotion. Therefore, medical schools need to regularly collect data on the patterns of tobacco use including alternative tobacco products as well. Medical students who smoke should be encouraged to seek cessation treatment and medical faculties need to offer cessation programs to help their students to quit. Information about the health effects of alternative tobacco products and cessation support for their users should also be provided. Currently none of the medical faculties participating in our study provide smoking cessation programs for students. Strengthening of tobacco-free university policies, as well as tobacco control laws within the countries where the medical schools are located and/or the countries from which medical students are from, may also contribute to a decrease in tobacco use among future physicians.

### Limitations

Despite the satisfactory response rate and a relatively large sample size, the real smoking prevalence may be higher, because smokers are usually overrepresented among non-responders [44]. Another limitation of this study is that it relies on self-reported smoking behavior. Furthermore, we had no data on other factors known to influence smoking status (peer pressure, history of parental smoking, etc.), which may explain the low coefficient of determination seen in our regression models. Recall bias and response bias could not be ruled out because data were self-reported. We sought to minimize social desirability bias, a common issue in surveys on sensitive topics, by assuring the anonymity of participants.

## Conclusions

Although the harm from tobacco use is undisputed, many medical students continue to smoke. Nevertheless, some positive trends were observed in smoking rates among future physicians in the recent years. We found a significant difference in smoking rates across different nations. There were fewer cigarette smokers among Norwegian students when compared to other countries. Prevalence of e-cigarette use was low, whereas waterpipe tobacco smoking was popular in our sample. Therefore, tobacco prevention and cessation programs for medical students should address not only the health risks of cigarette smoking but also discourage waterpipe use as well. Finally, medical schools should extend smoke-free areas from university buildings to the whole campus, which could have the dual advantage of creating a healthy environment and reducing the perceived social acceptance of smoking.

## Abbreviations

CI: Confidence interval
Exp(B): Exponentiation of the B coefficient
IR: Interquartile range
SD: Standard deviation
